# Hypoxia-Induced S100A8 Expression Activates Microglial Inflammation and Promotes Neuronal Apoptosis

**DOI:** 10.3390/ijms22031205

**Published:** 2021-01-26

**Authors:** Ji Sun Ha, Hye-Rim Choi, In Sik Kim, Eun-A Kim, Sung-Woo Cho, Seung-Ju Yang

**Affiliations:** 1 Department of Biomedical Laboratory Science, Konyang University, Daejeon 35365, Korea; jsha9595@gmail.com (J.S.H.); hr960625@naver.com (H.-R.C.); 2 Department of Biomedical Laboratory Science, School of Medicine, Eulji University, Uijeongbu 11759, Korea; orientree@hanmail.net; 3 Department of Biochemistry and Molecular Biology, University of Ulsan College of Medicine, Seoul 05505, Korea; kea0121@naver.com

**Keywords:** S100A8, microglia, inflammasome, hypoxia, neuronal apoptosis, COX-2

## Abstract

S100 calcium-binding protein A8 (S100A8), a danger-associated molecular pattern, has emerged as an important mediator of the pro-inflammatory response. Some S100 proteins play a prominent role in neuroinflammatory disorders and increase the secretion of pro-inflammatory cytokines in microglial cells. The aim of this study was to determine whether S100A8 induced neuronal apoptosis during cerebral hypoxia and elucidate its mechanism of action. In this study, we reported that the S100A8 protein expression was increased in mouse neuronal and microglial cells when exposed to hypoxia, and induced neuroinflammation and neuronal apoptosis. S100A8, secreted from neurons under hypoxia, activated the secretion of tumor necrosis factor (TNF-α) and interleukin-6 (IL-6) through phosphorylation of extracellular-signal-regulated kinase (ERK) and c-Jun N-terminal kinase (JNK) in microglia. Also, phosphorylation of ERK via the TLR4 receptor induced the priming of the NLRP3 inflammasome. The changes in Cyclooxygenase-2 (COX-2) expression, a well-known inflammatory activator, were regulated by the S100A8 expression in microglial cells. Knockdown of S100A8 levels by using shRNA revealed that microglial S100A8 expression activated COX-2 expression, leading to neuronal apoptosis under hypoxia. These results suggested that S100A8 may be an important molecule for bidirectional microglia-neuron communication and a new therapeutic target for neurological disorders caused by microglial inflammation during hypoxia.

## 1. Introduction

Stroke is the second leading cause of death and the major cause of disability worldwide [1]. Hypoxia, as one of the ischemic stroke-induced phenomena, was reported to contribute to neuroinflammation. Immediately after exposure to a hypoxic condition, neurons activate several processes that lead to inflammation, oxidative stress, excitotoxicity, and apoptosis [2,3,4]. The participation of other pathological mediators, including matrix metalloproteinases, high-mobility group box 1 (HMGB1), and mitogen-activated protein kinase (MAPK), could potentially perpetuate the ischemic brain damage [5]. As a result, the brain environment is exposed to systemic responses that further aggravate the immune responses and cause secondary neuronal damage.

Microglia are the principal brain-resident macrophages that rapidly respond to a variety of pathogens or damage, including ischemic stroke in the CNS [6,7]. Under brain ischemia, activated microglia initiate phagocytosis and the production of reactive oxygen species and inflammatory mediators, including interleukin (IL)-1β, tumor necrosis factor (TNF)-α, and interleukin (IL)-6 [8]. Various studies demonstrated that microglia-derived inflammatory and neurotoxic factors, such as TNF-α and IL-1β, elicited neuronal apoptosis [9,10]. In the activated microglia, HMGB1, a damage-associated molecular pattern (DAMP) protein, was actively secreted into the extracellular environment, leading to microglial activation and hippocampal neuronal apoptosis [11].

S100 calcium-binding protein A8 (S100A8), which belongs to the S100 family, mainly derives from immunocytes and participates in the inflammatory process. Many studies found that S100A8, a DAMP protein, promotes toll-like receptor (TLR)-4 activation in macrophages and plays an important role in a variety of neurodegenerative diseases [12]. During a stroke, the inflammatory process starts with the release of DAMPs from brain cells, which then activate microglia via TLRs [13,14]. High extracellular concentrations of S100A8 were observed in numerous inflammatory diseases, including Alzheimer’s disease, traumatic brain injury, and stroke [15,16]. S100A8/A9 stimulated the secretion of pro-inflammatory cytokines on LPS-induced BV-2 microglia cells via TLR4 and receptor for advanced glycation end products (RAGE) and exacerbated the damage of oligodendrocyte precursor cells by activating the nuclear factor (NF)-κB signaling pathway [17,18]. However, it remains unknown whether S100A8 plays a pivotal role in neuronal apoptosis.

Inflammasomes were recently discovered as multi-protein complexes that initiate immune responses associated with meningitis, stroke, and Alzheimer’s disease, including nucleotide-binding oligomerization domain-like receptor (NLR) and absent in melanoma 2 (AIM2)-like receptors [19]. The NLRP3 inflammasome function is regulated by two signaling pathways: ”priming” and ”activation”. During ”priming”, referred as signal 1, TLR induces the activation of the nuclear factor- κB (NF-κB) pathway to upregulate the expression of NLRP3 and pro-IL-1β proteins in various immune cells. NLRP3 inflammasome ”activation”, called also signal 2, is triggered by DAMPs to assemble the functional NLRP inflammasome, and thus activate the cleaved caspase-1 and IL-1β proteins [20,21]. Adenosine 5′-triphosphate disodium salt hydrate (ATP), which is present in the extracellular matrix of the cells, is a well-known DAMP signal and widely used for activation of the NLRP3 inflammasome. However, in a recent study, S100A8/A9 was also involved in inducing the signal 1 via the activation of NF-κB, mediated by reactive oxygen species, in human peripheral blood mononuclear cells [22]. Therefore, the mechanism by which S100A8 affected the signals of the NLRP3 inflammasome in ischemic microglia is unknown.

Cyclooxygenase-2 (COX-2) and prostaglandin E2 (PGE2) are well-known in vitro and in vivo inflammatory inducers [23]. Hypoxia and cellular injury upregulated the expression of COX-2 in human vascular endothelial and neuronal cells [24,25]. Neurotoxicity via elevating COX-2 expression was noted in neurodegenerative diseases, such as multiple sclerosis, amyotrophic lateral sclerosis, and Parkinson and Alzheimer diseases; meanwhile, the inhibition of COX-2 expression promoted neuroprotective effects, according to several experiments [26,27,28]. Hence, several selective COX-2 inhibitors have been developed but have not clearly shown anti-inflammatory effects in microglia. Therefore, the importance of designing a novel COX-2 activator and discovering its molecular mechanism in neuroinflammation is increasing.

Therefore, this study determined whether S100A8 induced neuronal apoptosis during cerebral hypoxia and elucidated its mechanism of action using in vitro systems, including astrocytes and microglial and neuronal cells, under hypoxic conditions.

## 2. Results

### 2.1. Hypoxia Increased the Production of S100A8 Protein in Neuronal and Microglial Cells and Induced the Secretion of S100A8 in SH-SY5Y Cells

**Figure 1:**
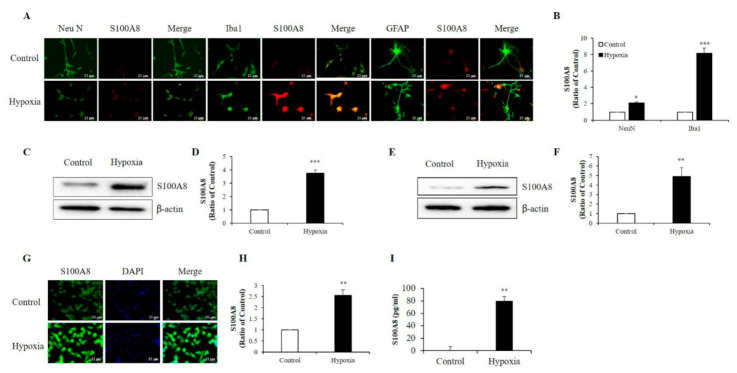
Hypoxia increased the production of S100 calcium-binding protein A8 (S100A8) in neuron and microglia and induced the release of S100A8 in SH-SY5Y cells. (**A**,**B**) S100A8 expression (red) were detected by immunocytochemical analysis in primary cultured neurons (NeuN, neuron marker) and cultured mixed glia (Iba1, microglial marker and GFAP, astrocyte marker) exposed to hypoxic conditions for 48 h. Scheme 25 μm. S100A8 expression was detected by western blot analysis in (**C**,**D**) SH-SY5Y cells and (**E**,**F**) BV-2 cells exposed to hypoxic conditions for 48 h. (**G**,**H**) S100A8 protein expression in BV-2 cells were confirmed by immunocytochemistry and (**I**) S100A8 release in SH-SY5Y was measured by enzyme-linked immunosorbent assay (ELISA) at 48 h after hypoxia. Values of * *p* < 0.05, ** *p* < 0.01, *** *p* < 0.001 versus control were considered as statistically significant.

S100A8 protein mainly derived from immunocytes, such as neutrophils and macrophages [12,22], and its expression was enhanced in the hippocampi of mouse models for amyloid-β overproduction [29]. To examine which cell type exhibited up-regulation of S100A8 expression after hypoxia (1% O_2_, 5% CO_2_, and 94% N_2_), we isolated and cultured neurons, microglia, and astrocytes from newborn mouse brains. Immunofluorescence showed that S100A8 expression was increased by about 2-fold in Neu N-positive primary neurons and about 8-fold in Iba1-positive primary microglia after exposure to a hypoxic condition; however, no obvious S100 expression in GFAP-positive primary astrocytes was detected (Figure 1A,B). Western blot analysis revealed a strong expression of S100A8 in SH-SY5Y neuronal (Figure 1C,D) and BV2 microglial (Figure 1E,F) cells under hypoxic conditions, when compared to that of control cells. The increase in S100A8 expression in BV2 cells was confirmed by immunocytochemistry (Figure 1G,H). Interestingly, the secretion of the S100A8 protein was detected in the culture supernatant of SH-SY5Y neuronal cells, but not in microglia (Figure 1I). The results showed that S100A8 levels were upregulated in neuronal and microglial cells and that neurons secreted the protein S100A8 under hypoxic conditions.

### 2.2. S100A8 Induced Pro-Inflammatory Cytokine Production Via Phosphorylation of ERK and JNK in BV-2 Cells

**Figure 2:**
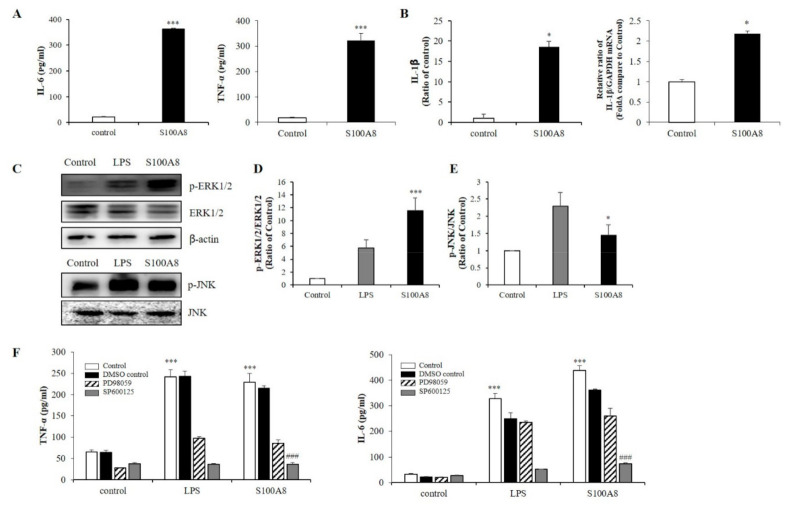
S100A8 induces pro-inflammatory cytokines and inflammation in BV-2 cells. BV-2 cells were stimulated with S100A8 (10 μg/mL) for 24 h. (**A**) The supernatant was collected and TNF-α and interleukin-6 (IL-6) analyzed by ELISA. (**B**) The protein and mRNA were extracted, and the expression levels of IL-1β were assessed by ELISA and RT-qPCR. (**C**–**E**) The protein was extracted, separated on 10% SDS-acrylamide gels (15 μg/lane) and transferred to nitrocellulose membrane. The protein expression level was detected by western blotting with anti-ERK1/2, anti-phospho-ERK1/2 (p-ERK1/2), anti-JNK and anti-p-JNK. (**F**) Cells were pre-treated with ERK inhibitor (PD98059, 20 μM), JNK inhibitor (SP600125, 10 μM) or the equivalent volume of DMSO for 1 h, then stimulated for 24 h with LPS or S100A8 for ELISA of TNF-α, IL-6. Data from three independent experiments are presented as the means ± S.D. Values of * *p* < 0.05, *** *p* < 0.001 versus control; ### *p* < 0.001 versus S100A8-treated sample were considered as statistically significant.

To measure the secretion of pro-inflammatory cytokines in microglia exposed to S100A8, BV-2 cells were treated with 10 μg/mL of S100A8 protein for 24 h. In S100A8-treated BV-2 cells, the secretion of interleukin-6 (IL-6) and TNF-α was greatly enhanced (Figure 2A) and the protein and mRNA expression of IL-1β was increased, compared to those of the control cells (Figure 2B). Also, the phosphorylation of c-Jun N-terminal kinase (JNK) and extracellular-signal-regulated kinase (ERK) were stimulated by S100A8, which had an analogous effect to the lipopolysaccharide (LPS) treatment (Figure 2C–E). PD98059 (ERK inhibitor) and SP600125 (JNK inhibitor) inhibited the S100A8-induced cytokine secretion (Figure 2F). These results suggested that S100A8 enhanced the levels of pro-inflammatory cytokines, such as IL-6 and TNF-α, via phosphorylation of ERK and JNK.

### 2.3. S100A8 Regulated NLRP3 Inflammasome Priming through TLR4/NF-κB Signaling in BV-2 Cells

**Figure 3:**
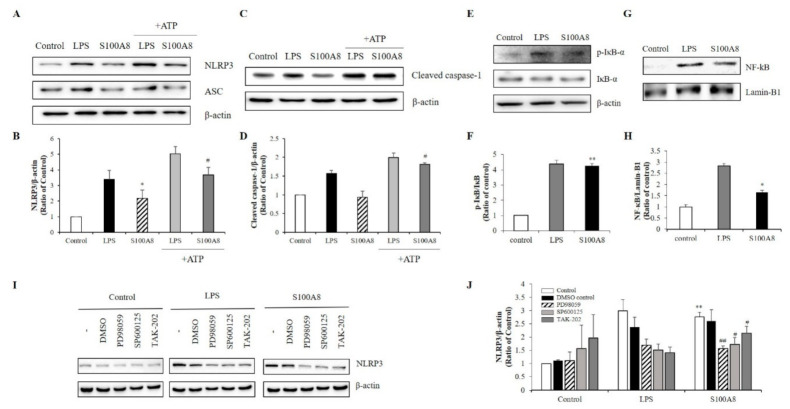
S100A8 induces inflammasome priming by toll-like receptor (TLR)-4 receptors associated with ERK and JNK pathway in BV-2 cells. BV-2 cells were incubated for 24 h with LPS (1 μg/mL) or S100A8 (10 μg/mL) followed by Adenosine 5′-triphosphate disodium salt hydrate (ATP) (1 mM) for 1 h. (**A**,**B**) The NLRP3, ASC, and (**C**,**D**) cleaved caspase-1 were detected by western blotting. β-actin was used as an internal control. (**E**,**F**) BV-2 cells were lysed to whole lysates and IκB-α phosphorylation was analyzed by western blotting. (**G**,**H**) The translocation of nuclear factor- κB (NF-κB) was also detected by western blotting. BV-2 cells were lysed to cytosolic extracts and nucleic extracts. Lamin-B1 was used as internal controls. (**I**,**J**) BV-2 microglial cells were pre-treated with PD98059 (ERK inhibitor, 20 μM), SP600125 (JNK inhibitor, 10 μM), TAK-202 (TLR4 inhibitor, 10 μg/mL) or an equivalent volume of DMSO and stimulated for 24 h with LPS or S100A8. Cells harvested and lysed in RIPA buffer for western blotting of NLRP3. Results are from one experiment that is representative of at least three others. Data from three independent experiments are presented as the means ± S.D. Values of * *p* < 0.05, ** *p* < 0.01 versus control; # *p* < 0.05, ## *p* < 0.01 versus S100A8-treated sample were considered as statistically significant.

During the priming of the NLRP3 inflammasome formation, caspase-1 levels are up-regulated, leading to an increase of pro-IL-1β concentration. In our study, the increase of IL-1β expression by S100A8 indicated that S100A8 was involved in the priming signal for NLRP3 inflammasome assembly in microglia (Figure 2B). Interestingly, the NLRP3 expression was increased about 2-fold following stimulation with S100A8 to levels comparable to those achieved by LPS stimulation and without affecting the expression of the adapter apoptosis-associated speck-like protein (ASC). The pretreatment of cells with S100A8, followed by stimulation with ATP, a known inflammasome activator, synergistically enhanced the NLRP3 and cleavage of caspase-1 about 2-fold, compared to that of control cells or cells primed with S100A8 alone (Figure 3A–D).

The NLRP3 inflammasome activation is controlled by a priming step that is dependent on the translocation of NF-κB into the nucleus [22]. Our results showed that phosphorylation of IκB-α, which played a critical role in activating NF-κB, increased in LPS- or S100A8-treated BV-2 cells (Figure 3E,F). Similarly, the translocation of NF-kB to the nucleus increased in cells treated with S100A8 (Figure 3G,H). These results strongly suggested that S100A8 induced the NLRP3 inflammasome priming via NF-κB activation. To elucidate the inflammasome priming mechanism, induced by S100A8, in BV-2 cells, we investigated how the NLRP3 inflammasome priming pathway was affected when using TAK-202 (TLR4 inhibitor), PD98059 (ERK inhibitor), and SP600125 (JNK inhibitor). Administration of TAK-202, PD98059, and SP600125 strongly inhibited the NLRP3 activation in cells stimulated with S100A8 or LPS (Figure 3I,J). The results obtained with the inhibitors suggested that the priming of NLRP3 inflammasome by S100A8 was associated with the ERK and JNK pathways via TLR4 receptors.

**Figure 4:**
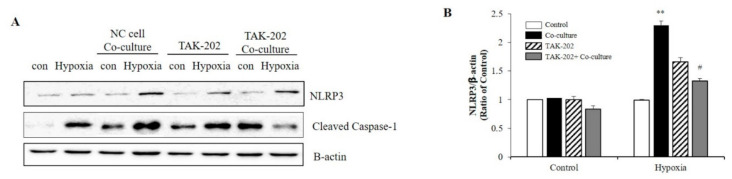
S100A8 derived from neuronal cells induces NLRP3 inflammasome priming in microglia under hypoxic conditions. BV-2 cells were pre-treated with TAK-202 (TLR4 inhibitor, 10 μg/mL) for 1 h, then stimulated for 48 h in hypoxic condition with SH-SY5Y cells indirectly co-cultured in 0.4 μm pore transwell. (**A**) The protein expression level was detected by western blotting with NLRP3. β-actin was used as an internal control. (**B**) Quantitative analysis of NLRP3 levels. Data from three independent experiments are presented as the means ± S.D. Values of ** *p* < 0.01 versus control; # *p* < 0.05 versus co-cultured sample were considered as statistically significant.

Subsequently, the present study evaluated whether S100A8, secreted from neurons under hypoxic conditions, was involved in microglial activation. Neuronal cells and microglia were co-cultured using transwells to confirm the NLRP3 inflammasome priming effects of S100A8 secreted by neuronal cells. Microglial cells were treated for 1 h with the TLR4 inhibitor, a receptor of S100A8, and co-cultivated for 48 h under hypoxic conditions. When co-cultured with the SH-SY5Y neuroblastoma cells under hypoxic conditions, the levels of NLRP3 and cleaved caspase-1 in BV-2 cells were significantly increased, but decreased after TAK-202 treatment (Figure 4A,B). The results suggested that S100A8, secreted by neuronal cells under hypoxic conditions, triggered the priming of NLRP3 in microglial cells, through the TLR4/NF-κB signaling.

### 2.4. S100A8 Knockdown on Microglia Attenuated Neuronal Apoptosis by Hypoxia

**Figure 5:**
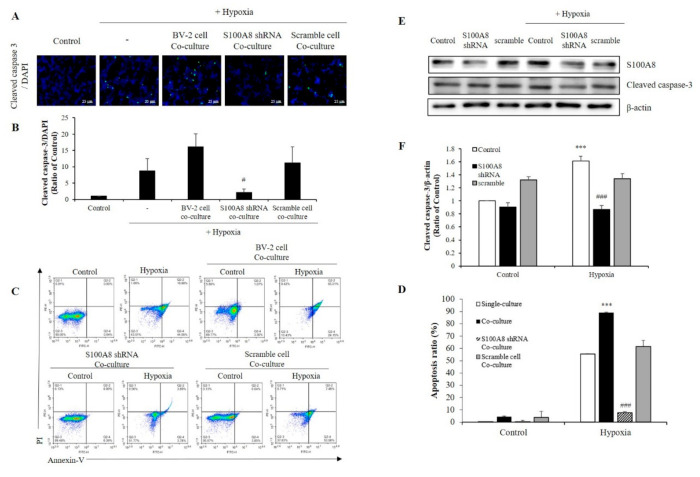
The expression of S100A8 in microglial cell induces apoptosis of neuronal cells in hypoxic condition. (**A**,**B**) SH-SY5Y cells incubated without or with S100A8 KD BV-2 cells for 48 h in hypoxic condition. Cleaved caspase-3 immunofluorescence images and were detected and quantitative analysis of the number of cleaved-caspase3-positive cells are shown in lower panel. (**C**,**D**) Representative Annexin-V/PI images were detected by flow cytometry. Quantitative analysis of the apoptotic rate of SH-SY5Y cells are shown in lower panel. (**E**,**F**) Primary neuron-glial mixed cells were transfected with S100A8 shRNA vector for 24 h followed by 48 h in hypoxic condition. Cells were harvested, and the expression protein levels of S100A8 and cleaved caspase-3 were analyzed by Western blotting. Data from three independent experiments are presented as the means ± S.D. Values of *** *p* < 0.001 versus control; # *p* < 0.05, ### *p* < 0.001 versus hypoxia-exposed sample were considered as statistically significant.

To investigate whether S100A8 expression in microglia induced apoptosis of neuronal cells under hypoxic condition, SH-SY5Y cells were co-cultured with BV-2 cells transfected with S100A8 shRNA for 48 h in a 0.4 μm pore transwell system and under hypoxic conditions (Figure 5A,B). In hypoxic condition, the concentration of cleaved caspase 3, an important mediator of apoptosis, was increased 25-fold when SH-SY5Y cells co--cultured with BV-2 cells, but this result was decreased approximately 8-fold in S100A8 shRNA transfected BV-2 cells. Flow cytometry analysis showed that SH-SY5Y apoptosis was significantly enhanced about 89-fold (annexin V-positive) when in presence of BV-2 cells under a hypoxic condition, and this effect was significantly suppressed 12-fold by S100A8 shRNA knockdown in BV-2 cells (Figure 5C,D). Moreover, in primary neuron-glial mixed cultures, the upregulation of cleaved caspase-3 was detected in the hypoxic conditions; however, transfection with shS100A8 decreased the cleaved caspase-3, compared with no transfection (Figure 5E,F). This result suggested that S100A8 expression in microglia affected the neuronal apoptosis.

**Figure 6:**
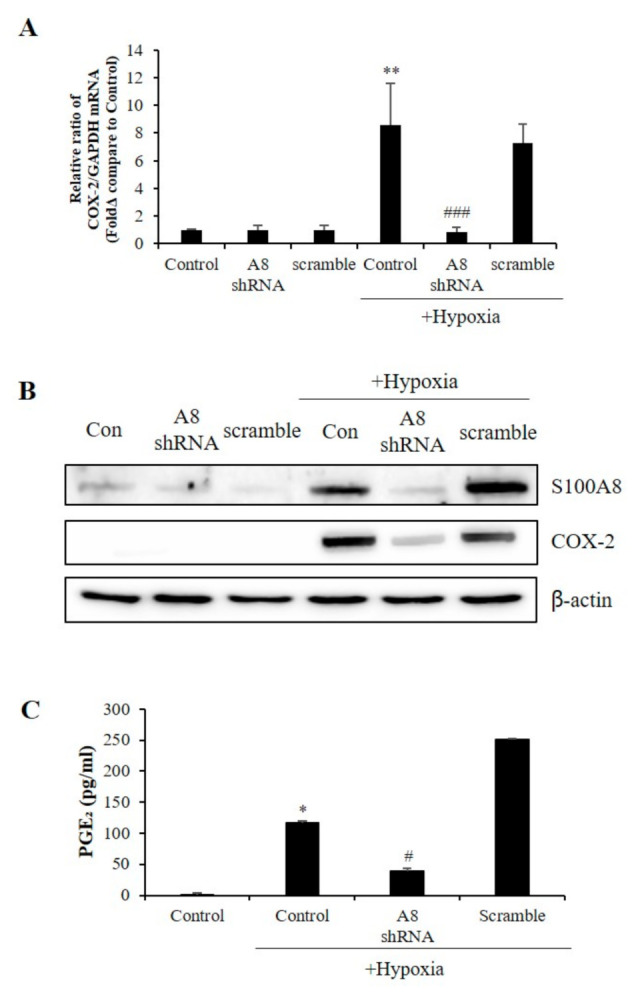
The expression of S100A8 in microglial cell induces the Cyclooxygenase-2 (COX-2)/prostaglandin E2 (PGE_2)_ pathway. BV-2 cells were transfected with S100A8 shRNA or Scramble vector. After 24 h, cells were incubated in hypoxic condition for 48 h. (**A**) The mRNA and (**B**) the protein levels of S100A8 and COX-2 were detected by real-time PCR and western blotting. (**C**) Secretion of PGE_2_ level analyzed by ELISA. Data from three independent experiments are presented as the means ± S.D. Values of * *p* < 0.05, ** *p* < 0.01 versus control; # *p* < 0.05, ### *p* < 0.001 versus hypoxia-exposed sample were considered as statistically significant.

COX-2 levels were increased in several cell types under acute and chronic inflammatory conditions [30]. Surprisingly, the reduction of COX-2 mRNA and protein levels correlated to that of S100A8 (Figure 6A,B). Also, hypoxic conditions significantly increased the secretion of PGE_2_; however, this effect was significantly inhibited by S100A8 shRNA (Figure 6C). These findings indicated that the expression of S100A8, induced in microglia cells under hypoxic conditions, activated COX-2 expression and PGE_2_ secretion to induce the apoptosis of neurons.

## 3. Discussion

DAMPs, such as HMGB1, S100, and heat shock proteins, have an important role in the inflammatory pathway of many neurodegenerative diseases and a variety of stresses. DAMPs are secreted by injured neurons and internal glial cells. Amyloid-β, known as the cause of Alzheimer’s disease (AD), is a representative DAMP molecule whose expression increases in glial cells and during neuroinflammation [31]. HMGB1 concentration significantly increased in AD patients, inducing inflammation and recruitment of immune cells in the peripheral blood [32]. In the ischemic brain, HMGB1 and peroxiredoxin family proteins are major DAMPs, but several more candidates (S100A8, S100A9, Mrp8, and Mrp9) have also been identified, although their relevance has not been clarified [13,14]. In 6- and 24-h post-ischemia of a mouse model of permanent middle cerebral artery occlusion, S100A8 mRNA was only expressed in the ischemic region [33]. Our results demonstrated that the increase of S100A8 protein expression was observed in mouse neuronal and microglial cells when exposed to hypoxia conditions, but not in mouse astrocytes. Interestingly, hypoxia induced the secretion of S100A8 by SH-SY5Y neuronal cells, and not by microglia. Therefore, S100A8 may be involved in the inflammatory communication between microglia and neuron during ischemia.

Microglial cells are immune cells in the central nervous system and play an important role in neuroinflammation in many brain diseases, including ischemic stroke [34,35]. Once ischemia occurs, microglia are activated, producing harmful mediators that are involved in the death of nerve cells [36]. TNF-α and IL-6 are pro-inflammatory cytokines secreted from activated microglia that communicate with neurons [37]. On the other hand, S100A8/A9 induced extensive activation of microglia and the production of multiple inflammatory factors, including TNF-α and IFN-γ [12]. Moreover, Wu reported that S100A8/A9 promoted BV-2 microglial cell activation, proliferation, migration, and polarization [18]. Pro-inflammatory cytokines are activated through MAPK pathways, including ERK and JNK, which are associated with neuronal cell death in diseases, such as AD, amyotrophic lateral sclerosis, and Parkinson’s disease [38]. In agreement with previous reports, the results of this study confirmed that S100A8 significantly increased the production of IL-6, TNF-α, and IL-1β. Meanwhile, S100A8 increased the phosphorylation levels of ERK and JNK in BV-2 cells; pro-inflammatory cytokine activities were decreased when cells were treated with PD98059 (ERK inhibitor) and SP600125 (JNK inhibitor). These results indicated that S100A8 activated the ERK and JNK pathways.

Molecular pathways leading to cytokine IL-1β secretion take place in intracellular complexes such as the NLRP3 inflammasome [39]. Recent research noted that NLRP3 was activated by priming signals from DAMPs associated with cellular stress, including extracellular ATP, amyloid-β, and HMGB1, initiating neuroinflammatory responses [40]. Also, NF-kB translocation was required for the activation of neuroinflammation and the establishment of NLRP3 inflammatory priming signals, that resulted in increased NLRP3 and procaspase-1 expression levels through TLR4 receptors [41]. DAMPs are frequently associated with activation of NLRP3 inflammasome but in some cases DAMPs may also be able to act as signal 1 to prime NLRP3 [21]. However, in the absence of an initial priming stimulus, DAMPs have no effect on the expression of IL-1β in mouse mixed glia [42]. Surprisingly, we confirmed that S100A8 regulated the NLRP3 inflammasome priming, which increased NLRP3 and IL-1β expression in BV-2 cells. S100A8/A9 proteins have the capacity to prime the inflammasome, as shown using in vitro peripheral blood mononuclear cells [22]. However, our results demonstrated for the first time the importance of S100A8 in the upregulation of NLRP3 in microglial cells. In addition, the translocation of NF-kB, which played a pivotal role in regulating the expression and activation of NLRP3, was also increased when cells were treated with S100A8. However, the NLRP3 priming effect of S100A8 was reduced by treatment with PD98059 (ERK inhibitor), SP600125 (JNK inhibitor), and TAK-202 (TLR4 inhibitor). Given that the levels of secreted S100A8 protein increased in the mouse neurons when exposed to hypoxia, we considered that the intracellular signals, induced by S100A8, triggered the activation of the NLRP3 inflammasome and the expression of inflammatory cytokines. Indeed, NLRP3 was activated when co-cultured with SH-SY5Y cells under hypoxic conditions but its activation markedly decreased when treated with TAK-202. These results suggested that S100A8, secreted by neuronal cells under hypoxic conditions, combined with TLR4 of microglia cells, activated the NLRP3 inflammasome priming.

Some pathogens, protein aggregates, or cytokines may activate microglia; meanwhile, inflammatory stimuli could induce them to express and release TNF-α and cathepsin B, possibly resulting in neuronal apoptosis [9]. Overproduction of TNF-α after oxygen-glucose deprivation induced microglial activation and later neuronal apoptosis [10]. HMGB1, secreted by activated microglia, promoted the apoptosis of hippocampal neurons [11]. S100A8/A9-induced BV-2 microglial cell activation, promoting the apoptosis of oligodendrocyte precursor cells [18]. Our study showed that hypoxia increased the production of S100A8 in microglia. In our transwell co-culture system, we found that cleaved caspase 3 expression further increased in SH-SY5Y cells when co-cultured with BV-2 cells but decreased in the presence of S100A8 shRNA-transfected BV-2 cells under hypoxic conditions. FACS analysis showed that the increase of S100A8 levels in microglia by hypoxia promoted neuronal apoptosis, which was confirmed by immunofluorescence. Indeed, apoptosis of SH-SY5Y cells was increased to 90% when co-cultured with BV-2 cells in comparison to when cultured alone, but significantly decreased by 10% when co-cultured with S100A8 KD BV-2 cells under hypoxic conditions. Co-culture experimental results would not explain in vivo neural network between neurons and microglial cells. However, for the first time, we showed that up-regulation of microglial S100A8 levels increased neuronal apoptosis after hypoxia, in primary multicellular cultures consisting of neurons, astrocytes, and microglia.

COX-2 catalyzes the synthesis of prostaglandins in the metabolism of arachidonic acid and is rapidly expressed in many cell types in response to growth factors, cytokines, and pro-inflammatory molecules. Overexpression of COX-2 indicates nerve damage after various acquired injuries, and thus, many COX-2 inhibitors suppressed various pathways [42,43,44]. The S100A8 knockdown using shRNA revealed that COX-2 and PGE_2_ expression was regulated by S100A8, which suggested that the intracellular increase of microglial S100A8 levels upregulated COX-2 expression and PGE2 secretion, contributing to neuronal death under hypoxic conditions. However, more research is required to determine whether S100A8 directly regulates COX-2.

## 4. Materials and Methods

### 4.1. Materials

Dulbecco’s modified Eagle’s medium (DMEM) and phosphate-buffered saline (PBS) were provided by Corning (New York, NY, USA). Penicillin (100 U/mL), streptomycin (100 μg/mL), heat-inactivated fetal bovine serum (FBS), Hank’s Balanced Salt Solution (HBSS), and D-glucose were purchased from Gibco (Life Technologies Inc., Gaithersburg, MD, USA). Adenosine 5′-triphosphate disodium salt hydrate (ATP) was obtained from Sigma-Aldrich (St. Louis, MO, USA). The S100A8 protein was synthesized and purified as previously described [45]. S100A8, TNF-α, IL-6, IL-1β and PGE2 were quantitatively measured by an enzyme-linked immunosorbent assay (ELISA) using the human S100A8 Duoset ELISA kits, the mouse TNF-α, IL-6 and IL-1β DuoSet ELISA kits, and the PGE2 parameter assay kit (R&D systems, Minneapolis, MN, USA), according to the manufacturer’s instructions. The antibodies used for western blot were: anti-p-ERK1/2 (42, 44 kDa, Thr-202 and Thr-204), anti-ERK1/2 (42, 44 kDa), anti-NLRP3 (110 kDa), anti-p-IκB-α (39 kDa), anti-p-IκB-α (40 kDa, Ser-32), anti-NFκB (65 kDa), anti-COX2 (74 kDa), anti-rabbit IgG HP and anti-mouse IgG HP from Cell Signaling Technology (Danvers, MA, USA); anti-S100A8 (11 kDa), anti-NeuN, anti-Iba1, and anti-GFAP, anti-cleaved caspase-3 (17 kDa) from Abcam (Cambridge, UK); and, anti-p-JNK (46, 54 kDa, Ser-63 and Ser-73 residues), anti-JNK (46, 54 kDa), anti-ASC (24 kDa), anti-caspase-1 (p20) (45/20 kDa), anti-Lamin-B1 (67 kDa), and anti-β-actin (43 kDa) from Santa Cruz Biotechnology (Dallas, TX, USA). TLR 4 (TAK-202), JNK (SP600125), and ERK inhibitors (PD98059) were provided by R&D systems (Minneapolis, MN, USA).

### 4.2. Cell Culture

Primary neuron-glial mixed cultures were obtained from the hippocampus of 3-day-old newborn ICR mouse pups (Koatech, South Korea). Briefly, hippocampi were dissected and digested with 1 X HBSS buffer, 1 M HEPES, 3 g glucose, and penicillin/streptomycin. Then, the dissociated cells were immediately seeded onto flasks and incubated in high glucose DMEM containing 10% FBS, penicillin (100 U/mL), and streptomycin (100 μg/mL) for 24 h in a CO_2_ incubator. The media was changed every three days until the 12th day. Immunofluorescent staining identified nerve cells, microglial cells, and astrocytes by using the anti-NeuN (Abcam, Cambridge, UK), anti-ionized calcium-binding adapter molecule 1 (Iba1, Abcam, Cambridge, UK), and anti-GFAP primary antibodies (Abcam, Cambridge, UK).

BV-2 and SH-SY5Y cells were obtained from the Department of Biochemistry and Molecular Biology, University of Ulsan College of Medicine (Seoul, South Korea) and were maintained in DMEM supplemented with penicillin (100 U/mL), streptomycin (100 μg/mL), and 10% FBS at 37 °C. The cells were incubated in a humidified atmosphere containing 5% CO_2_. The hypoxic condition consisted of exposing the cells to 1% O_2_, 5% CO_2_, and 94% N_2_ in a hypoxic chamber (Stemcell Technologies, Vancouver, BC, Canada) for 24 or 48 h.

BV-2 and SH-SY5Y cells were indirectly co-cultured in a 0.4 μm pore transwell system (Corning, New York, NY, USA) or with supernatants of monocultures.

### 4.3. Plasmids and Transfection

Lentiviral GFP vectors containing the S100A8 shRNA or scrambled shRNA sequence (5′-AACTTGTTCAGAGAATTGGACATCAATAG-3′) were purchased from Origene (Rockville, MD, USA). Cells were transfected with S100A8 shRNA or scramble shRNA using Lipofectamine 3000 reagent (Invitrogen, Waltham, Massachusetts, USA) in a serum-reduced medium, according to the manufacturer’s instructions. After 6 h, the medium was changed to complete medium, and the cells were incubated for another 24 h before performing other experiments.

### 4.4. Measurement of Apoptosis by Flow Cytometry

To confirm whether the apoptotic effect in the microglial cells was caused by S100A8, apoptosis was detected using Alexa Fluor^®^ 488 Annexin V/Dead Cell Apoptosis kit, according to the manufacturer’s protocol. Briefly, after exposure to hypoxia for 48 h, SH-SY5Y cells were harvested and washed twice with cold PBS. The cells were then suspended in 100 µL Annexin-binding buffer and stained by adding 5 µL Annexin V-fluorescein isothiocyanate and 1 µL of 100 µg/mL PI solution for 15 min in the dark at room temperature. Next, 400 µL of Annexin-binding buffer was added to each sample. Finally, apoptotic levels were analyzed by flow cytometry (NOVOCYTE flow cytometer, ACEA Biosciences Inc, San Diego, CA, USA). Data were analyzed using the Novoexpress software (ACEA Biosciences Inc, San Diego, CA, USA).

### 4.5. Western Blot Analysis

BV2 cells, SH-SY5Y cells, and primary cells were lysed using RIPA buffer (Thermo Scientific, Waltham, MA, USA), and the protein concentrations were determined using the Lowry protein assay. Thereafter, samples with an equal amount of protein were separated by sodium dodecyl sulfate (SDS)-polyacrylamide gel electrophoresis on 10–12% SDS gels. The proteins were transferred onto a nitrocellulose membrane (Bio-rad, Hercules, CA, USA), and then the membrane was incubated overnight at 4 °C with the primary antibodies, followed by incubation for 1 h at room temperature with either the anti-rabbit IgG HRP or the anti-mouse IgG HRP secondary antibody. The protein bands on the membrane were developed using an enhanced chemiluminescence detection system (Vilber Lourmat, Collégien, France).

### 4.6. ELISA

The levels of the cytokines, such as S100A8, TNF-α, IL-6 and PGE_2_ (R&D systems, Minneapolis, MN, USA) were detected with ELISA kits, according to the manufacturer’s instructions. In brief, cultured cell supernatants were collected following various treatments. The absorbance of each solution was measured at 450 nm using a microplate reader. All assays were performed as three independent experiments. The levels of cytokines were calculated using the standard value obtained from a linear regression equation.

### 4.7. Immunofluorescence Analysis

The cells were cultured on coverslips and treated as indicated before. Then, the cells were fixed with 4% paraformaldehyde at room temperature for 10 min and permeabilized with 0.25% Triton X-100 at room temperature for 10 min. The unspecific binding of antibodies to the cells was blocked by incubating the cells with 1% BSA and 22.52 mg/mL glycine in 0.1% PBST solution for 1 h at room temperature. Next, cells on the coverslips were incubated overnight with a primary antibody at 4 °C and then the corresponding secondary antibody at room temperature for 1 h. The nuclei were counterstained with DAPI. Finally, the stained cells were observed using ZOE™ Fluorescent Cell Imager (Bio-Rad, Hercules, CA, USA)

### 4.8. Real-Time Quantitative PCR Analysis

After treating the cells under hypoxic conditions for 48 h or in presence of S100A8 protein for 24 h, cells were collected using Trizol reagent (Life technologies, Carlsbad, CA, USA) and lysed, according to the manufacturer’s instructions. Then, the extracted RNA was quantified using the NanoDrop^TM^ One, and the cDNA was synthesized by using the DiaStarTM 2X RT Pre-Mix. Real-time PCR was conducted to quantify the cDNA by using the CFX96TM real-time system and the SsoAdvanced^TM^ Universal SYBR Green Supermix. Furthermore, the following primers were used: IL-1β Forward (F): 5′-GCCCATCCTCTGTGACTCAT-3′, Reverse (R): 5′-AGGCCACAGGTAT TTTGTCG-3′, S100A8 F: 5′-GGAAATCACCATGCCCTCTA-3′, R: 5′-TGGCTGTCTTTGTGAGATGC -3′, GAPDH F: 5′-TCACCACCATGGAGAAGGC-3′, R: 5′-GCTAAGCAGTTGGTGGTGCA-3′.

### 4.9. Statistical Analyses

Data are presented as mean ± standard error (SD) and represent the average of three independent experiments. The SPSS statistical software package (Version 18.0, USA) was used for the analysis of variance (ANOVA), as appropriate. Additionally, the differences between the groups were compared with the one-way ANOVA, followed by Scheffe and Dunnett T3 methods. The results with *p*-values < 0.05 were considered statistically significant.

## 5. Conclusions

This study demonstrated the role of S100A8 on microglia at intracellular and extracellular levels under hypoxic conditions. The intracellular concentration of S100A8 molecules in the microglia as well as the levels of S100A8 secreted from the neurons affected the neuroinflammation under hypoxic conditions. S100A8 secreted from neurons activated ERK, JNK, and the priming signals of NLRP3 inflammasome through TLR4 receptors in microglial cells. In addition, S100A8 constituted a novel COX-2 activator in microglia during neuroinflammation, indicating that S100A8 may be a new regulator of neuroinflammation under hypoxic conditions, which occurs in ischemic stroke.

## Data Availability

Not applicable.

## Abbreviations

DAMP	Damage-associated molecular pattern
ERK	Extracellular-signal-regulated kinase
JNK	c-Jun N-terminal kinase
HMGB1	High-mobility group box 1
S100A8	S100 calcium-binding protein A8
TLR4	Toll-like receptor-4
MAPK	Mitogen-activated protein kinase
NLR	Nucleotide-binding oligomerization domain-like receptor
COX-2	Cyclooxygenase-2
PGE2	Prostaglandin E2
IL-6	Interleukin-6
TNF-α	Tumor necrosis factor
RAGE	Receptor for advanced glycation end products
NF-κB	Nuclear factor- κB
AIM2	Absent in melanoma 2
ASC	Adapter apoptosis-associated speck-like protein
AD	Alzheimer’s disease
DMEM	Dulbecco’s modified Eagle’s medium
PBS	Phosphate-buffered saline
FBS	Fetal bovine serum
HBSS	Hank’s Balanced Salt Solution
ATP	Adenosine 5′-triphosphate disodium salt hydrate
SDS	Sodium dodecyl sulfate

## References

[1] Johnson W., Onuma O., Owolabi M., Sachdev S. (2016). Stroke: A global response is needed. Bull. World Heal. Organ..

[2] Lee Y., Lee S., Park J.-W., Hwang J.-S., Kim S.-M., Lyoo I.K., Lee C.-J., Han I.-O. (2018). Hypoxia-Induced Neuroinflammation and Learning–Memory Impairments in Adult Zebrafish Are Suppressed by Glucosamine. Mol. Neurobiol..

[3] Khoshnam S.E., Winlow W., Farzaneh M., Farbood Y., Moghaddam H.F. (2017). Pathogenic mechanisms following ischemic stroke. Neurol. Sci..

[4] Lo E.H., Dalkara T., Moskowitz M.A. (2003). Mechanisms, challenges and opportunities in stroke. Nat. Rev. Neurosci..

[5] Jayaraj R.L., Azimullah S., Beiram R., Jalal F.Y., Rosenberg G.A. (2019). Neuroinflammation: Friend and foe for ischemic stroke. J. Neuroinflamm..

[6] Bok S., Kim Y.E., Woo Y., Kim S., Kang S.J., Lee Y., Park S.K., Weissman I.L., Ahn G.O. (2017). Hypoxia-inducible factor-1α regulates microglial functions affecting neuronal survival in the acute phase of ischemic stroke in mice. Oncotarget.

[7] Greenhalgh A.D., David S., Bennett F.C. (2020). Immune cell regulation of glia during CNS injury and disease. Nat. Rev. Neurosci..

[8] Tsuji S., Di Martino E., Mukai T., Tsuji S., Murakami T., Harris R.A., Blomgren K., Åden U. (2020). Aggravated brain injury after neonatal hypoxic ischemia in microglia-depleted mice. J. Neuroinflamm..

[9] Guadagno J., Xu X., Karajgikar M., Brown A., Cregan S. (2013). Microglia-derived TNF α induces apoptosis in neural precursor cells via transcriptional activation of the Bcl-2 family member Puma. Cell Death Dis..

[10] Zhang L., Dong L.-Y., Lian-Yan D., Hong Z., Wei W.-S. (2012). The microRNA miR-181c controls microglia-mediated neuronal apoptosis by suppressing tumor necrosis factor. J. Neuroinflamm..

[11] Shi Y., Guo X., Zhang J., Zhou H., Sun B., Feng J. (2018). DNA binding protein HMGB1 secreted by activated microglia promotes the apoptosis of hippocampal neurons in diabetes complicated with OSA. Brain Behav. Immun..

[12] Wang S., Song R., Wang Z., Jing Z., Wang S., Ma J. (2018). S100A8/A9 in Inflammation. Front. Immunol..

[13] Shichita T., Ito M., Yoshimura A. (2014). Post-ischemic inflammation regulates neural damage and protection. Front. Cell. Neurosci..

[14] Gülke E., Gelderblom M., Magnus T. (2018). Danger signals in stroke and their role on microglia activation after ischemia. Ther. Adv. Neurol. Disord..

[15] Li B., Concepcion K., Meng X., Zhang L. (2017). Brain-immune interactions in perinatal hypoxic-ischemic brain injury. Prog. Neurobiol..

[16] Xia C., Braunstein Z., Toomey A.C., Zhong J., Rao X. (2018). S100 Proteins As an Important Regulator of Macrophage Inflammation. Front. Immunol..

[17] Ma L., Sun P., Zhang J.C., Zhang Q., Yao S.L. (2017). Proinflammatory effects of S100A8/A9 via TLR4 and RAGE signaling pathways in BV-2 microglial cells. Int. J. Mol. Med..

[18] Wu M., Xu L., Wang Y., Zhou N., Zhen F., Zhang Y., Qu X., Fan H., Liu S., Chen Y. (2018). S100A8/A9 induces microglia activation and promotes the apoptosis of oligodendrocyte precursor cells by activating the NF-κB signaling pathway. Brain Res. Bull..

[19] Walsh J.G., Muruve D.A., Power C. (2014). Inflammasomes in the CNS. Nat. Rev. Neurosci..

[20] Jo E.-K., Kim J.K., Shin D.-M., Sasakawa C. (2016). Molecular mechanisms regulating NLRP3 inflammasome activation. Cell. Mol. Immunol..

[21] Fann D.Y.-W., Lim Y.-A., Cheng Y.-L., Lok K.-Z., Chunduri P., Baik S.-H., Drummond G.R., Dheen S.T., Sobey C.G., Jo D.-G. (2018). Evidence that NF-κB and MAPK signaling promotes NLRP inflammasome activation in neurons following ischemic stroke. Mol. Neurobiol..

[22] Simard J.-C., Cesaro A., Chapeton-Montes J., Tardif M., Antoine F., Girard D., Tessier P.A. (2013). S100A8 and S100A9 induce cytokine expression and regulate the NLRP3 inflammasome via ROS-dependent activation of NF-κB 1. PLoS ONE.

[23] Williams C.S., Mann M., Dubois R.N. (1999). The role of cyclooxygenases in inflammation, cancer, and development. Oncogene.

[24] Bonazzi A., Mastyugin V., Mieyal P.A., Dunn M.W., Laniado-Schwartzman M. (2000). Regulation of Cyclooxygenase-2 by Hypoxia and Peroxisome Proliferators in the Corneal Epithelium. J. Biol. Chem..

[25] Aïd S., Bosetti F. (2011). Targeting cyclooxygenases-1 and -2 in neuroinflammation: Therapeutic implications. Biochimie.

[26] Minghetti L. (2004). Cyclooxygenase-2 (COX-2) in Inflammatory and Degenerative Brain Diseases. J. Neuropathol. Exp. Neurol..

[27] Xia Q., Hu Q., Wang H., Yang H., Gao F., Ren H., Chen D., Fu C., Ying Z., Zhen X. (2015). Induction of COX-2-PGE2 synthesis by activation of the MAPK/ERK pathway contributes to neuronal death triggered by TDP-43-depleted microglia. Cell Death Dis..

[28] Sánchez-Pernaute R., Ferree A., Cooper O., Yu M., Brownell A.-L., Isacson O. (2004). Selective COX-2 inhibition prevents progressive dopamine neuron degeneration in a rat model of Parkinson’s disease. J. Neuroinflamm..

[29] Lodeiro M., Puerta E., Ismail M.-A.-M., Rodriguez-Rodriguez P., Rönnbäck A., Codita A., Parrado-Fernandez C., Maioli S., Gil-Bea F., Merino-Serrais P. (2017). Aggregation of the inflammatory S100A8 precedes Aβ plaque formation in transgenic APP mice: Positive feedback for S100A8 and Aβ productions. J. Gerontol. Ser. A.

[30] Vijitruth R., Liu M., Choi D.-Y., Nguyen X.V., Hunter R.L., Bing G. (2006). Cyclooxygenase-2 mediates microglial activation and secondary dopaminergic cell death in the mouse MPTP model of Parkinson’s disease. J. Neuroinflamm..

[31] Clark I.A., Vissel B. (2015). Amyloid β: One of three danger-associated molecules that are secondary inducers of the proinflammatory cytokines that mediate A lzheimer’s disease. Br. J. Pharmacol..

[32] Fujita K., Motoki K., Tagawa K., Chen X., Hama H., Nakajima K., Homma H., Tamura T., Watanabe H., Katsuno M. (2016). HMGB1, a pathogenic molecule that induces neurite degeneration via TLR4-MARCKS, is a potential therapeutic target for Alzheimer’s disease. Sci. Rep..

[33] Hori M., Nakamachi T., Rakwal R., Shibato J., Nakamura K., Wada Y., Tsuchikawa D., Yoshikawa A., Tamaki K., Shioda S. (2012). Unraveling the ischemic brain transcriptome in a permanent middle cerebral artery occlusion mouse model by DNA microarray analysis. Dis. Models Mech..

[34] Szepesi Z., Manouchehrian O., Bachiller S., Deierborg T. (2018). Bidirectional microglia–neuron communication in health and disease. Front. Cell. Neurosci..

[35] McDonough A., Lee R.V., Noor S., Lee C., Le T., Iorga M., Phillips J.L., Murphy S., Möller T., Weinstein J.R. (2017). Ischemia/reperfusion induces interferon-stimulated gene expression in microglia. J. Neurosci..

[36] Zhao S.C., Ma L.S., Chu Z.H., Xu H., Wu W.Q., Liu F. (2017). Regulation of microglial activation in stroke. Acta Pharmacol. Sin..

[37] Hanisch U.-K. (2002). Microglia as a source and target of cytokines. Glia.

[38] Kim E.K., Choi E.-J. (2010). Pathological roles of MAPK signaling pathways in human diseases. Biochim. Biophys. Acta (BBA) Mol. Basis Dis..

[39] Gustin A., Kirchmeyer M., Koncina E., Felten P., Losciuto S., Heurtaux T., Tardivel A., Heuschling P., Dostert C. (2015). NLRP3 inflammasome is expressed and functional in mouse brain microglia but not in astrocytes. PLoS ONE.

[40] Mangan M.S., Olhava E.J., Roush W.R., Seidel H.M., Glick G.D., Latz E. (2018). Targeting the NLRP3 inflammasome in inflammatory diseases. Nat. Rev. Drug Discov..

[41] He Y., Hara H., Núñez G. (2016). Mechanism and regulation of NLRP3 inflammasome activation. Trends Biochem. Sci..

[42] Lee S., Shin S., Kim H., Han S., Kim K., Kwon J., Kwak J.-H., Lee C.-K., Ha N.-J., Yim D. (2011). Anti-inflammatory function of arctiin by inhibiting COX-2 expression via NF-κB pathways. J. Inflamm..

[43] Zarghi A., Arfaei S. (2011). Selective COX-2 Inhibitors: A Review of Their Structure-Activity Relationships. Iran. J. Pharm. Res..

[44] Xing Y., Wang R., Chen D., Mao J., Shi R., Wu Z., Kang J., Tian W., Zhang C. (2015). COX2 is involved in hypoxia-induced TNF-α expression in osteoblast. Sci. Rep..

[45] Kim D.H., Choi E., Lee J.-S., Lee N.R., Baek S.Y., Gu A., Kim D.H., Kim I.S. (2015). House Dust Mite Allergen Regulates Constitutive Apoptosis of Normal and Asthmatic Neutrophils via Toll-Like Receptor 4. PLoS ONE.

